# Meropenem vs standard of care for treatment of late onset sepsis in children of less than 90 days of age: study protocol for a randomised controlled trial

**DOI:** 10.1186/1745-6215-12-215

**Published:** 2011-09-30

**Authors:** Irja Lutsar, Ursula MT Trafojer, Paul T Heath, Tuuli Metsvaht, Joseph Standing, Susanna Esposito, Vincent Meiffredy de Cabre, Clarissa Oeser, Jean-Pierre Aboulker

**Affiliations:** 1 Institute of Microbiology, University of Tartu, Ravila 19, Tartu, 50411, Estonia; 2 Neonatal Intensive Care Unit, Department of Pediatrics, Via Giustiniani 3, Padova, 35128, Italy; 3 Division of Clinical Sciences, St George's, University of London, Cranmer Terrace, Londo, SW17 0RE, UK; 4 Clinic of Anaesthesiology and Intensive Care, University Clinics of Tartu, Lunini 6, Tartu, 50411, Estonia; 5 Infectious Diseases and Microbiology Unit, Institute of Child Health University College London, 30 Guilford Street, London, WC1N 1EH, UK; 6 Department of Maternal and Pediatric Sciences, Università degli Studi di Milano, Fondazione IRCCS Ca' Granda Ospedale Maggiore Policlinico, Via Commenda 9, Milano, 20122, Italy; 7 INSERM SC10, 16, avenue Paul-Vaillant Couturier, Villejuif, 94807, France; 8 Via Giustiniani 3, 35128, Padova, Italy

**Keywords:** randomised controlled trial, neonate, premature neonate, FP7

## Abstract

**Background:**

Late onset neonatal sepsis (LOS) with the mortality of 17 to 27% is still a serious disease. Meropenem is an antibiotic with wide antibacterial coverage. The advantage of it over standard of care could be its wider antibacterial coverage and thus the use of mono-instead of combination therapy.

**Methods:**

NeoMero-1, an open label, randomised, comparator controlled, superiority trial aims to compare the efficacy of meropenem with a predefined standard of care (ampicillin + gentamicin or cefotaxime + gentamicin) in the treatment of LOS in neonates and infants aged less than 90 days admitted to a neonatal intensive care unit.

A total of 550 subjects will be recruited following a 1:1 randomisation scheme. The trial includes patients with culture confirmed (at least one positive culture from normally sterile site except coagulase negative staphylococci in addition to one clinical or laboratory criterion) or clinical sepsis (at least two laboratory and two clinical criteria suggestive of LOS in subjects with postmenstrual age < 44 weeks or fulfilment of criteria established by the International Pediatric Sepsis Consensus Conference in subjects with postmenstrual age ≥ 44 weeks). Meropenem will be given at a dose of 20 mg/kg q12h or q8h depending on the gestational- and postnatal age. Comparator agents are administered as indicated in British National Formulary for Children. The primary endpoint measured at the test of cure visit (2 days after end of study therapy) is graded to success (all baseline symptoms and laboratory parameters are resolved or improved with no need to continue antibiotics and the baseline microorganisms are eradicated and no new microorganisms are identified and the patient has received allocated treatment for 11 ± 3 days with no modification) or a failure (all remaining cases). Secondary outcome measures include comparison of survival, relapse rates or new infections by Day 28, clinical response at Day 3 and end of therapy, duration of hospitalisation, population pharmacokinetic analysis of meropenem and effect of antibiotics on mucosal colonisation and development of antibacterial resistance.

The study will start recruitment in September 2011; the total duration is of 24 months.

**Trial registration:**

EudraCT 2011-001515-31

## Background

A recent review including eight studies conducted in European and Australian neonatal wards showed that 80 to 93% of prescriptions are for *off-label *or unlicensed products [1]. In its 7^th ^framework programme the European Union has funded several projects that specifically aim to evaluate pharmacokinetics (PK), efficacy and safety of off-patent medicines in children. One project funded in 2010 is NeoMero-1, an open label, randomised, controlled superiority trial that aims to compare the efficacy of meropenem with a predefined standard of care (SOC) for the treatment of late onset sepsis (LOS) in neonates and infants aged less than 90 days (inclusive) admitted to a neonatal intensive care unit (NICU). The safety of study regimens as well as LOS-causing microorganisms and their antibiotic susceptibility patterns, bacterial eradication-, relapse- and superinfection rates, mucosal colonisation with antibiotic resistant microorganisms and fungi, genetic markers that may affect response to therapy and the PK characteristics of meropenem will be described as secondary outcome measures.

### Late onset sepsis

LOS is commonly defined as sepsis occurring 48 to 72 hours after birth, and is a serious disease with a mortality rate of 17% to 27% depending on the studied population [2-5]. Although LOS is predominantly caused by coagulase negative staphylococci (CoNS) accounting for 36-66% of cases [3,4,6-8], Gram-negative rods *Klebsiella pneumoniae*, *Escherichia coli*, *Serratia *spp and *Enterobacter cloacae *are responsible for about 26-36% of cases [4,6,9]. In recent years the incidence of Gram negative infections appears to be increasing in many NICUs worldwide [10-12]. In an UK based microbiology laboratory survey, after exclusion of CoNS, the majority of LOS isolates were susceptible to the commonly used combinations of empiric antibiotics flucloxacillin, gentamicin, ampicillin and/or cefotaxime, however, relatively high resistance rates of *Enterobacteriaceae *to cephalosporins were reported [3,13].

### Use of meropenem in neonates

Meropenem is a low protein-bound (2%) broad-spectrum carbapenem with the bactericidal activity resulting from inhibition of bacterial peptidoglycan synthesis in the cell wall. It is active against Gram-positive and Gram-negative bacteria including anaerobes and extended spectrum beta-lactamase and AmpC chromosomal β-lactamase producing *Enterobacteriaceae *[14]. The pharmacodynamics (PD) of meropenem is determined by the fraction of time free drug exceeds MIC (f%T>MIC) with the target value of about 70% suggested for immunosuppressed adults and potentially for neonates [15].

The PK of meropenem in neonates has been described in three studies [16-18] but firm dosing recommendations have not yet been made. By using Monte Carlo simulations (MCS) two studies [16,18] concluded that for most microorganisms a dose of 10 mg/kg or 20 mg/kg given twice or three times a day, depending on the gestational (GA) and postnatal age (PNA), is sufficient since >90% of treated neonates will achieve PK/PD target attainment. One possible short-coming of the simulation approach taken by Bradley *et al *[18] was that MIC values were randomly allocated to simulated patients, thereby assuming MICs were known *a priori*, and many of the allocated MIC values were likely well below the accepted breakpoints for sensitivity. When designing dosing regimens, MIC is not known in advance and it is therefore preferable to recommend doses that will cover all organisms up to the sensitivity breakpoint which is usually 2 mg/L for meropenem (http://www.eucast.org). This approach was taken by van den Anker *et al*. [16] and in fact both authors emphasized that higher doses and 4-hour infusion may be needed for microorganisms with increased MIC values, more specifically for *Pseudomonas aeruginosa *[16,18]. However, this may not be feasible due to the instability of meropenem (Summary of Product Characteristics). Whilst it is recommended that meropenem should be given within 1 hour of reconstitution, a recent study has shown that at a concentration of 4% at room temperature (≤ 25°C) the degradation will be less than 10% over 12 hours [19].

The advantage of meropenem over standard of care might be its wider antibacterial coverage and thus the use of mono- instead of combination therapy.

### Clinical trials in LOS

Despite the fact that a number of clinical studies in neonatal sepsis have been conducted previously, several methodological issues and the changing patient population make their results out of date and not applicable to the present population affected by LOS. It is remarkable that all 13 randomised controlled trials (RCT) are relatively old, dating from 1973 to 1992 [20]. With the exception of two studies, [21,22] data on EOS and LOS were combined and neonatal and paediatric patients were reported together [23,24]. The variable proportion of missing outcomes as well as the small sample sizes in the two trials which included only LOS patients makes drawing meaningful conclusions from these studies also difficult. Our proposed NeoMero1 study is therefore designed to address many of these gaps in the literature.

## Methods and Design

### Eligible patients

**Figure 1:**
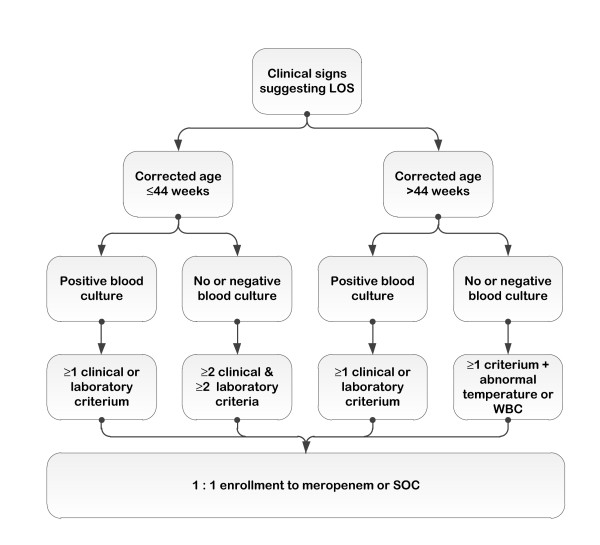
**Flowchart of the NeoMero1 study**.

The study will include patients with confirmed as well as clinical sepsis (Figure 1). Confirmed sepsis will be defined as at least one positive bacterial culture (except CoNS) from a normally sterile site at baseline together with an abnormal clinical or laboratory parameter within the last 24 hours prior to randomisation (as listed below). Clinical sepsis in patients equal or older than 44 weeks of postmenstrual age is based on the criteria defined by the International Pediatric Sepsis Consensus Conference [25]. For patients below 44 weeks of postmenstrual age, the criteria of clinical sepsis were agreed at the Expert Meeting on Neonatal and Paediatric Sepsis on 8 June 2010, EMA London (http://www.ema.europa.eu/docs/en_GB/document_library/Report/2010/12/WC500100199.pdf) and consist of a combination of at least two abnormal clinical and two abnormal laboratory parameters within the 24 hours prior to randomisation. The relevant clinical criteria are (1) hyper- or hypothermia or temperature instability; (2) reduced urinary output or hypotension or mottled skin or impaired peripheral perfusion; (3) apnea or increased oxygen or increased ventilatory support requirement; (4) bradycardia spells or tachycardia or rhythm instability; (5) feeding intolerance or abdominal distension; (6) lethargy or hypotonia or irritability; (7) skin and subcutaneous lesions such as petechial rash or sclerema. The relevant laboratory criteria are: (1) white blood cells count (WBC) < 4 or > 20 × 10^9 ^cells/L; (2) immature to total neutrophil (I/T) ratio > 0.2; (3) platelet count < 100 × 10^9^/L; (4) C-reactive protein (CRP) > 15 mg/L or procalcitonin ≥ 2 ng/mL; (5) glucose intolerance when receiving normal glucose amounts (8-15 g/kg/day) as expressed by blood glucose values > 180 mg/dL or hypoglycemia (< 40 mg/dL) confirmed on at least two occasions; (6) acidosis with base excess (BE) < -10 mmol/L or lactate above 2 mmol/L.

Patients who have received systemic antibiotics for more than 24 hours (except when judged as treatment failure), have organisms suspected or known to be resistant to study therapies or are not expected to survive for more than 3 months due to congenital abnormalities, will be excluded as well as patients with renal failure or known intolerance or contraindications to the study medications.

### Study treatments

**Figure 2:**
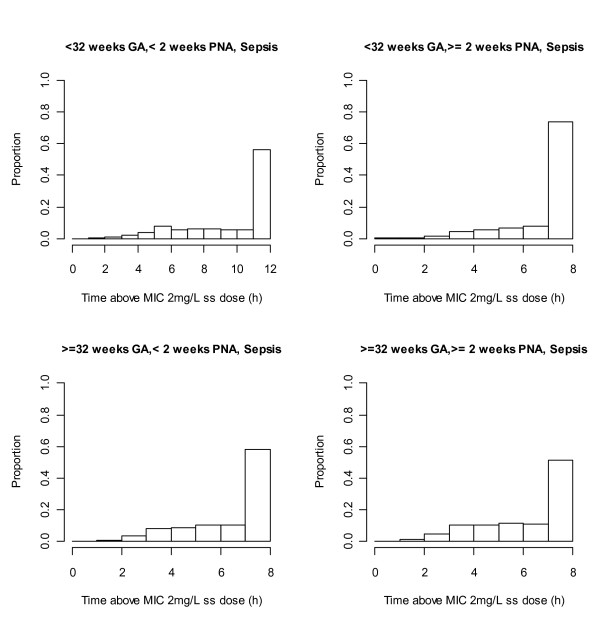
**Histograms denoting simulated steady-state T>MIC for subjects receiving infusions of meropenem**. Each chart presents different group of subjects depending on GA and PNA. The T>MIC was calculated based on the EUCAST breakpoints for *Enterobacteraciae *which is 2 mg/L. PNA - postnatal age, GA - gestational age; ss - steady state

Meropenem dose for this study was set at 20 mg/kg q8h (q12h for neonates with GA < 32 weeks and PNA <2 weeks) based on the results of a previous PK study [18]. The main question to be addressed was infusion length, as giving the same dose but varying the infusion length will change f%T>MIC without affecting the area under the curve (AUC) and therefore overall exposure [16]. Using PK parameters and their uncertainty from an ongoing study in the USA [unpublished interim model kindly provided by Dr EV Capparelli], we performed simulations to investigate the effect of infusion length on f%T>MIC using the current EUCAST susceptibility cut-off for *Enterobacteriaceae *of 2 mg/L (http://www.eucast.org). These simulations showed that infusion times of 0.5, 1 and 2 hours gave 90% of patients with f%T>MIC of at least 44%, 48%, and 50%, respectively with a skewed distribution meaning that at least 60% in all age groups had f%T>MIC of 100% (Figure 2). Clearly increasing infusion length increased f%T>MIC, but given concerns over meropenem's stability and that at least 44% f%T>MIC was achieved for 90% of patients, a 30 minute infusion was finally recommended.

The two most commonly used antibiotic regimens for LOS (ampicillin + gentamicin or cefotaxime + gentamicin) were selected as SOC knowing that both regimens cover the vast majority of LOS causing agents and have been in use for several decades [13]. To minimise the potential bias of an open label design, each participating unit will select just one of these two regimens prior to start of the study and will then use it throughout the study. The dosages for the comparator agents were selected as recommended in the BNFC (edition of 2010-2011; http://www.bnfc.org).

The use of other systemic antibacterials will not be allowed with the exception of vancomycin and other suitable antibiotics for the treatment of infections proven or suspected to be caused by methicillin-resistant CoNS or *Staphylococcus aureus*. The use of topical anti-infectives, systemic antifungals, antivirals, immunglobulins and probiotics is allowed and will be recorded in the case report form.

### Ethical aspects

Since the treatment of LOS is an emergency situation requiring immediate commencement of antibacterial treatment it will not always be possible to obtain the informed consent (IC) prior to the first dose of any antibiotic. Therefore the study will allow the enrolment of patients who have been given antibiotics for LOS according to the local guidelines for less than 24 hours. This avoids restricting the study to the less severe cases where treatment can be delayed until IC has been signed. For the study analysis patients will be stratified according to whether or not they received prior antibiotics.

The study aims to minimise interventions made purely for study purposes. First, all study antibiotics (including meropenem) are widely used for the treatment of LOS in the participating units despite the fact that none of them is labelled for neonates [26]. Second, few procedures will be conducted only for study purposes; PK samples, genetic samples and specific microbiologic tests will preferably be collected when routine clinical sampling is done; the collection of stool samples does not cause any extra distress to the baby/infant. Furthermore, the amount of blood collected for the PK-study (0.3 ml on a maximum of 3 times) has proven to be safe for ELBW babies [27].

The study has been approved by the Ethics Review Committee University of Tartu (205T-18).

### Monitoring of clinical and laboratory parameters

In addition to routine clinical monitoring, patients will be assessed for specific clinical signs and symptoms and for laboratory abnormalities at baseline, on Day 3, at the end of the antibacterial- and study treatment and in patients with a successful outcome, two days after stopping study treatments (test of cure visit - TOC). At all time points, clinical symptoms and laboratory parameters will be graded and compared with those at baseline. They will be categorised as improved, stable or worsened according to prespecified criteria. For monitoring relapses, reinfections, new infections and late safety issues a Day 28 follow up visit will be conducted for all patients with a successful outcome at TOC visit. This follow-up (FU) visit is optional and may be replaced by a telephone interview if necessary in patients with treatment failure. An auditory assessment will also be undertaken at any time up until the FU assessments. Finally, a separate study will invite patients at the corrected age of 20 to 24 months to neurodevelopmental and auditory testing if the latter was abnormal in the acute period.

### Microbiological assessments

Microbiological samples will be collected at baseline and on Day 3 and also whenever clinically indicated; they will be processed in local microbiology laboratories. Clinically significant isolates from blood or CSF will be stored at -80°C and sent for identification and antibacterial susceptibility to a central microbiology laboratory in order to ensure uniformity.

To compare the effect of study antibiotics on colonisation by antibiotic resistant microorganisms and fungi, stool samples or perianal swabs will be collected on admission, at the end of study therapy and at hospital discharge or the follow up visit (whichever is earliest). For a combination of techniques such as the detection of 16S ribosomal DNA [28], organism specific PCRs and microarray techniques [29,30], 0.5 mL of whole blood will be taken on admission and again on Day 3. Molecular techniques will be used to improve the detection of pathogens where conventional bacterial cultures are negative.

### Selection of optimal time points for PK sampling

Using the previously mentioned interim PK model parameters and their uncertainty, we undertook an ED-Optimal design exercise to ascertain when to take samples given the following scenarios: a small cohort of full profile subjects with 3 post-dose samples, and the remaining subjects providing a single PK sample. Optimal sampling times were found to be 0.5, 7-8 and 12 hours post dose for those dosed 12 hourly and 0.5, 5-6 and 8 hours post-dose for those dosed 8 hourly. For the single sample subjects, the optimal time was a trough sample.

### Statistical aspects

We opted for an efficacy trial. The superiority of meropenem over SOC is mainly expected from its wider coverage of responsible pathogens and from its potential use as monotherapy. We estimate that, in the control arm, 15% of patients will die before the TOC visit and among those who survive the proportion of failing subjects will be 25% [4,31]. Thus, the proportion of neonates who will die or fail therapy in the comparator arm is expected to be 36.25%. It is hypothesized that in the meropenem arm the survival will be improved to 90% and the failure rate will drop to 15% in surviving neonates. Thus, the expected proportion of neonates who will die or fail therapy should be reduced to 23.5%. Based on these assumptions, the required sample size to provide 80% power to show the superiority of meropenem over SOC, using a continuity-corrected chi-square test with a two-sided 5% alpha level, is 220 subjects per arm (NQuery software). We also anticipate that 15 to 20% of randomised neonates will ultimately have conditions not amenable to study antibiotics, although fulfilling the initial criteria of clinical sepsis, and thus decrease the apparent treatment size effect. Consequently, the sample was conservatively increased by 25%, thus the total population of the study will be 275 neonates per study arm.

The primary endpoint is a combination of clinical and microbiological criteria measured at the TOC visit and defined to a success (the patients is alive, all baseline symptoms and laboratory parameters are resolved or improved so that there is no need to continue antibacterial treatment, the baseline microorganisms are eradicated, there are no new microorganisms identified and patient has received allocated treatment for 11 ± 3 days with no modification) and a failure (all remaining cases).

Primary efficacy analysis will compare success rates in the two arms in all randomised subjects in an intent to treat approach. Subsidiary analyses of the primary endpoint by the stratification factors (SOC regimen, timing of antibiotic therapy initiation) will also be performed in the full analysis set and the confirmed sepsis subset. Various secondary analyses including comparison of survival and relapse rates or new infections by Day 28, clinical response at Day 3 and end of therapy visits, clinical and bacteriological response and duration of hospital stay will also be evaluated.

## Discussion

The evidence base for novel anti-infective treatments is limited in newborns, despite their high-risk status for infection and their vulnerability to its consequences. To our best knowledge, this is the first randomised controlled study in neonates and young infants with LOS conducted in the modern era of neonatal care. Although it is generally accepted that with antibiotics efficacy data in paediatric patients may not be needed as it might be extrapolated from adults, neonates are an exception [32]. It is possible that due to the immaturity of the immune system, the required PK/PD targets in critically ill neonates differ from those in adults, but we are not aware of these targets having been established [15].

A major challenge for all studies concerning LOS is the lack of validated clinical and laboratory criteria in situations where obtaining blood cultures is difficult, sample volumes are small, a substantial number of cultures could be negative or contaminated, and almost all clinical symptoms are non-specific. While the International Pediatric Sepsis Consensus Conference criteria [25] could be used for infants and neonates of postmenstrual age of 44 weeks and above, validated criteria for premature babies, accounting for the majority of LOS cases, are almost completely lacking. Modi *et al*. [33] have developed a predictive model of 12 predefined clinical criteria for LOS and suggested that the presence of three or more clinical symptoms has the best predictive accuracy for positive blood culture in premature neonates. Adding increased CRP into the model reduced the accuracy; this is not surprising as CRP is a late rather than early marker of infection. Others have shown that laboratory markers such as a platelet count < 100,000/ml [34], procalcitonin concentration > 2.3-2.4 ng/ml [35,36] or CRP >10 mg/L [37] are beyond normal ranges in premature babies. Evaluation of clinical symptoms is often subjective and could potentially introduce additional bias especially in a multicenter and international setting. So we have agreed on a combination of at least two clinical symptoms and at least two laboratory parameters to define clinical sepsis. We hypothesize that using these criteria the number of patients not having LOS will be minimised. Fairly similar criteria for LOS were used by one of the NeoMero Consortium partners in a previous study [38] and were also agreed by the EMA expert panel in June 2010 (http://www.ema.europa.eu/docs/en_GB/document_library/Report/2010/12/WC500100199.pdf).

To describe the PK/PD properties of meropenem, a population PK (popPK) analysis approach will be used as this allows a description of the variability in PK parameters and their dependence on patient characteristics. PopPK also allows the pooling of sparse data in many subjects in order to make predictions of f%T>MIC and its variability given a model derived from the data; the use of this technique for antimicrobial studies is increasingly recommended [39]. We have been able to inform the study design through simulation, in this case to define infusion length, and optimal PK sampling [40]. We have ensured that maximal information can be gained from the minimally invasive sampling schedule that has necessarily been imposed due to resource, logistical and patient characteristics in the proposed study. Whilst the recommended infusion time of 30 minutes does not achieve our original target of f%T>MIC of 70%, it has been suggested that f%T>MIC of 40% is sufficient for the bactericidal activity of carbapenems in patients with a functioning immune system [39]. Furthermore, our simulations present a worst-case scenario, in that many of the microorganisms we expect to encounter will have MIC values significantly lower than the EUCAST breakpoints. Through the use of population PK/PD modelling and MCS, the model created from the extensive data collected in this study will determine the optimal dose and infusion duration of meropenem.

An unresolved issue in neonatal studies, especially when dealing with patients in critical conditions or requiring rapid therapeutic interventions, is obtaining written IC. Several aspects were considered by the NeoMero Consortium. We appreciate that obtaining an IC prior to randomisation could be extremely challenging. It is expected that the vast majority of potential participants will be severely ill babies and the parents may not be available to sign the IC straight away. Although not proven in neonates, significant evidence from adult studies supports the role of immediate appropriate antibacterial treatment in reducing mortality in severe sepsis or septic shock [41]. Therefore in this study the following options to obtain IC will be explored in agreement with local regulations and Ethics Committees' position. First, written IC for potential LOS cases will be obtained around the time of the NICU admission. The downside of this option involves unnecessary consent in many patients and a possible change in the parent's opinion on participation with the sublimation of the status from a well-baby to a baby with a potentially life threatening illness. On the other hand, this procedure guarantees the parents a longer cooling-off period and offers more possibilities to seek further information before signing the consent form. The other option would be to consent over the phone and ask for signed consent when parents are next in the hospital. A pragmatic option is therefore to seek consent at the time of the study procedures where possible but also, in recognition that the study objectives will not be significantly affected, to obtain consent even after other antibiotic treatment has been commenced. None of these strategies is ideal and the preference will also depend on local requirements. This issue is not specific to the NeoMero studies and might be one of the reasons that no studies have been conducted in neonatal sepsis over the last 20 years despite the fact that most antibiotics in neonates are used off-label [26].

We appreciate that the ideal study design would be a blinded rather than an open label study. However, due to various antibiotic susceptibilities of pathogens in participating countries and differences in medical practices, finding a single acceptable comparator regimen appeared too complicated. Blinding would further be compromised by the fact that meropenem monotherapy is to be compared with a combination of comparator agents (ampicillin or cefotaxime plus gentamicin). A dummy infusion in critically ill, premature babies might lead to fluid overload and result in deterioration of the babies' condition. In an attempt to minimise potential bias the study sites have to commit to a specific comparator regimen prior to initiation of the study rather than at the time of randomisation, as this be influenced by their perception of the severity of the baby's illness.

Another controversial issue is the influence of meropenem or other broad spectrum antibiotics on the intestinal microflora. Some studies have suggested that broad spectrum antibiotics interfere with the development of intestinal microflora via outselection of antibiotic resistant microorganisms which may persist for several months [42,43]. Others, however, show that selection of resistant organisms is not associated with broad spectrum antibiotics but rather with the duration of the NICU stay and the presence of indwelling catheters [44,45]. This study provides an opportunity to assess the influence of different regimens on intestinal microflora in a controlled setting.

## Conclusions

Patients are planned to be randomised from September 2011. The results of the study should provide useful data on efficacy, safety and PK/PD of meropenem in the treatment of LOS in neonates and infants up to 90 days of age. In addition, data about immunogenetics of LOS as well as the distribution of causative agents and their antimicrobial resistance patterns will be provided. By providing a comprehensive list of prospectively collected data the study will also fulfil an important gap in our knowledge of neonatal sepsis. The data could then be used for further dose modelling for other antibiotics and likely allow more appropriate extrapolations of efficacy data from adult studies.

## Competing interests

The authors declare that they have no competing interests.

## Authors' contributions

IL and UT are principle investigators of the study, participated in the design of the study and drafted the manuscript; TM and PTH, participated in the design of the study and drafted the manuscript; CO and SE participated in the design of the study; JFS designed the pharmacokinetic part of the study and drafted relevant parts in the manuscript; VMC and JPA participated in the design of the study and performed statistical analysis. All authors read and approved the final manuscript.
